# Sustainability in Health care by Allocating Resources Effectively (SHARE) 2: identifying opportunities for disinvestment in a local healthcare setting

**DOI:** 10.1186/s12913-017-2211-6

**Published:** 2017-05-05

**Authors:** Claire Harris, Kelly Allen, Richard King, Wayne Ramsey, Cate Kelly, Malar Thiagarajan

**Affiliations:** 1 School of Public Health and Preventive Medicine, Monash University, Victoria, Australia; 2 Centre for Clinical Effectiveness, Monash Health, Victoria, Australia; 3 Medicine Program, Monash Health, Victoria, Australia; 4 Medical Services and Quality, Monash Health, Victoria, Australia; 5 Medical Services, Melbourne Health, Victoria, Australia; 6 Ageing and Aged Care Branch, Department of Health and Human Services, Victoria, Australia

**Keywords:** Disinvestment, Decommission, De-adopt, De-list, De-implement, Health Clinical Practice, TCP, Resource allocation, Decision-making, Implementation

## Abstract

**Background:**

This is the second in a series of papers reporting a program of Sustainability in Health care by Allocating Resources Effectively (SHARE) in a local healthcare setting. Rising healthcare costs, continuing advances in health technologies and recognition of ineffective practices and systematic waste are driving disinvestment of health technologies and clinical practices that offer little or no benefit in order to maximise outcomes from existing resources. However there is little information to guide regional health services or individual facilities in how they might approach disinvestment locally. This paper outlines the investigation of potential settings and methods for decision-making about disinvestment in the context of an Australian health service.

**Methods:**

Methods include a literature review on the concepts and terminology relating to disinvestment, a survey of national and international researchers, and interviews and workshops with local informants. A conceptual framework was drafted and refined with stakeholder feedback.

**Results:**

There is a lack of common terminology regarding definitions and concepts related to disinvestment and no guidance for an organisation-wide systematic approach to disinvestment in a local healthcare service.

A summary of issues from the literature and respondents highlight the lack of theoretical knowledge and practical experience and provide a guide to the information required to develop future models or methods for disinvestment in the local context.

A conceptual framework was developed. Three mechanisms that provide opportunities to introduce disinvestment decisions into health service systems and processes were identified. Presented in order of complexity, time to achieve outcomes and resources required they include 1) Explicit consideration of potential disinvestment in routine decision-making, 2) Proactive decision-making about disinvestment driven by available evidence from published research and local data, and 3) Specific exercises in priority setting and system redesign.

**Conclusion:**

This framework identifies potential opportunities to initiate disinvestment activities in a systematic integrated approach that can be applied across a whole organisation using transparent, evidence-based methods. Incorporating considerations for disinvestment into existing decision-making systems and processes might be achieved quickly with minimal cost; however establishment of new systems requires research into appropriate methods and provision of appropriate skills and resources to deliver them.

**Electronic supplementary material:**

The online version of this article (doi:10.1186/s12913-017-2211-6) contains supplementary material, which is available to authorized users.

## About SHARE

*This is the second in a series of papers reporting a program of Sustainability in Health care by Allocating Resources Effectively (SHARE). The SHARE Program is an investigation of concepts, opportunities, methods and implications for evidence-based investment and disinvestment in health technologies and clinical practices in a local healthcare setting. The papers in this series are targeted at clinicians, managers, policy makers, health service researchers and implementation scientists working in this context. This paper discusses potential settings and methods to initiate disinvestment decisions in an Australian health service network.*

## Background

In the past two decades, proactive and explicit methods have been sought to address rising healthcare costs and continuing advances in expensive health technologies. This has coincided with increasing recognition of ineffective practices and systemic waste in health services. As a result, debate and research has focused on removal of health technologies and clinical practices that offer little or no benefit in order to maximise outcomes from existing resources and the concept of ‘disinvestment’ has emerged [1, 2]. Cessation of potentially harmful, clinically ineffective or cost-inefficient procedures has the dual advantage of improving patient care and allowing for more efficient use of available resources, potentially increasing total health benefits without increasing spending.

In their 2007 paper, Pearson and Littlejohns considered the options available to the National Institute for Health and Clinical Effectiveness (NICE) to provide guidance and direction on disinvestment to the English National Health Service [1]. They explored the role of an agency that has both the imprimatur to lead the debate and the resources to enable informed decision-making at the national level. Development of national policies and production of rigorous evidence-based guidance are crucial steps, but there are other complex issues to be addressed before disinvestment can be successful across the whole health sector.

Decisions to allocate resources can be made at macro (national, state/provincial and regional), meso (institutional) and micro (individual) levels [3]; but even those made centrally still need to be implemented locally. In addition, some decisions cannot be made centrally as national recommendations cannot take into account local factors such as population needs, organisational priorities, budgets, capacity or capability. Hence many essential decisions about the use of health technologies and clinical practices (TCPs), programs and services are made at regional and institutional levels [4]. However, there is little information to guide regional health authorities or local facilities in how they might take a systematic approach to disinvestment [5–14]. The approach taken by Pearson and Littlejohns to guide disinvestment efforts at the national level can be adapted to inform decision-making at the local health service level [1].

Leaders at Monash Health (previously Southern Health), a large health service network in Melbourne, Australia, sought to establish an organisation-wide, systematic, integrated, transparent, evidence-based approach to disinvestment. This became known as the SHARE Program, exploring ‘Sustainability in Health care by Allocating Resources Effectively’ and was undertaken by the Centre for Clinical Effectiveness (CCE), an in-house resource to facilitate Evidence Based Practice (EBP). An overview of the SHARE Program, a guide to the SHARE publications and further details about Monash Health and CCE are provided in the first paper in this series [15].

**Fig. 1:**
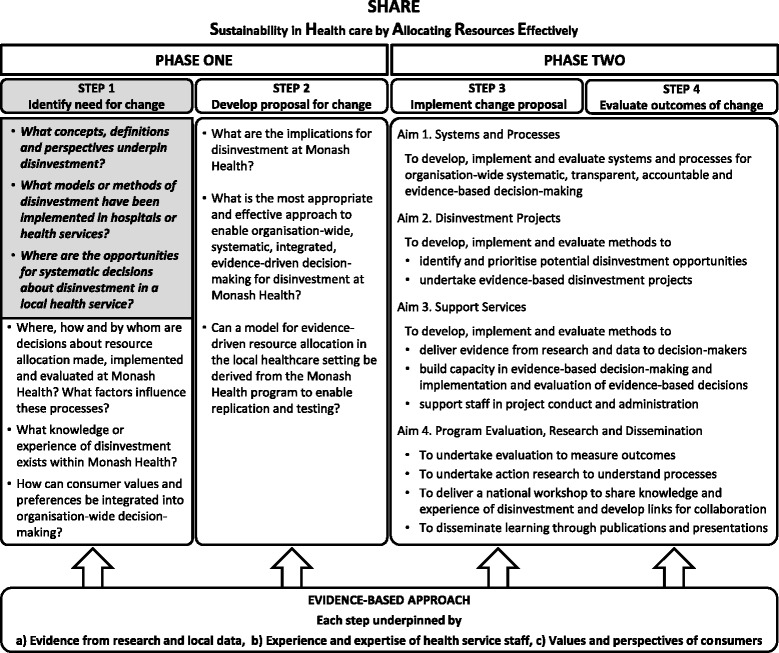
Overview of SHARE Program

In the absence of guidance from the literature, a two-phased process was proposed to identify and then evaluate potential opportunities for disinvestment at Monash Health (Fig. 1). The aim of Phase One was to understand concepts and practices related to disinvestment and the implications for a local health service and, based on this information, to identify potential settings and methods for decision-making. The aim of Phase Two was to implement and evaluate the proposed methods to determine which were sustainable, effective and appropriate at Monash Health.

### Aims

The aim of this project was to identify opportunities for systematic decisions about disinvestment at Monash Health.

The aim of this paper is to report the investigation of potential settings and methods for disinvestment decision-making and propose a framework to integrate them into local health service systems and processes.

### Research questions

What concepts, definitions and perspectives underpin disinvestment?

What models or methods of disinvestment have been implemented in hospitals or health services?

Where are the opportunities for systematic decisions about disinvestment in a local health service?

## Methods

### Model for evidence-based change

The SHARE Program was undertaken using the SEAchange model for Sustainable, Effective and Appropriate change in health services [16]. The model involves four steps: identifying the need for change, developing a proposal to meet the need, implementing the proposal and evaluating the extent and impact of the change. Each step is underpinned by the principles of evidence-based practice to ensure that the best available evidence from research and local data, the experience and expertise of health service staff and the values and perspectives of consumers are taken into account. Steps 1 and 2 of the SEAchange model map to Phase One of the SHARE Program and Steps 3 and 4 correspond to Phase Two. The research questions for this paper are highlighted in Fig. 1.

### Data collection

A literature review, interviews with members of the Technology/Clinical Practice Committee (TCPC) that initiated the SHARE Program, a survey of external experts, and workshops with the SHARE Steering Committee were conducted. Interviews with key local informants were undertaken to discuss the findings from the earlier activities and obtain additional information. Details are provided in Additional file 1.

### Development of the framework

Findings from the literature review, survey, workshops and interviews were collated and analysed thematically by either content analysis [17] to identify emergent themes, or framework analysis [18] when categories had been specified a priori (Additional file 1). The potential settings and methods identified were drafted into a conceptual framework.

This was presented to the SHARE Steering Committee for feedback and decision-making (Additional file 1). The committee included Executive Directors (Medical, Nursing, Support Services), Program Directors (Medical, Nursing, Allied Health, Pharmacy, Diagnostic Services), Committee chairs (Technology/Clinical Practice, Therapeutics, Human Research and Ethics, Clinical Ethics), Managers (Information Services, Clinical Information Services, Procurement, Biomedical Engineering, Research Services), Legal counsel and two Consumer representatives.

The CCE project team had expertise in EBP and knowledge brokering. This expertise contributed to committee discussions regarding evidence-based decision-making and implementation and evaluation of change.

Discussion was informal and decisions were based on consensus.

The framework was refined based on input from the committee and the project team.

## Results and Discussion

Results of the literature search and response rates and representativeness of participants in the survey, workshops and interviews are included in Additional file 1. The full literature review is published separately [19]. Surveys were received from 15 external experts, 13 members of the Steering Committee attended the workshops and 10 individuals participated in interviews.

Data collected from these activities informed a range of research questions. Findings related to the research questions in this paper are presented below and discussed in the context of the current literature; additional findings are reported in other SHARE publications.

### What concepts, definitions and perspectives underpin disinvestment?

The literature review identified a lack of common terminology and noted that several terms were used to describe disinvestment-type activities such as ‘decommissioning’, ‘removing ineffective services’, ‘resource release’ and ‘defunding’.

There were also multiple definitions for ‘disinvestment’ which were underpinned by different concepts (Table 1). Some definitions considered disinvestment to be reallocation of resources from one TCP to another while others were limited to removal or restriction of use without reference to reallocation. Some definitions were based on the relative value of one TCP over an alternative such as ‘this TCP has less value than that TCP’. Others were based on assessment of the absolute value of a TCP for example ‘this TCP is not worth funding’.

**Table 1:** Examples of concepts underpinning disinvestment definitions

Concept	Definition
Reallocation based on Relative value	“Disinvestment is an explicit process of taking resources from one service in order to use them for other purposes that are believed to be of better value” (Pearson and Littlejohns 2007 [1])
Reallocation based on Absolute value	“Disinvesting in health interventions that offer no or low health gain (eg are unproven, outdated or cost ineffective) provides an opportunity to invest in alternative proven and cost effective health interventions” (Victorian Department of Human Services 2007 [85])
Removal or restriction based on Absolute value	“Disinvestment relates to the withdrawing (partially or completely) of health care practices, procedures, technologies and pharmaceuticals that are deemed to deliver no or low health gain and are thus not efficient or appropriate health resources allocations” (Elshaug et al. [2])

Reasons for disinvestment were similarly based on a range of concepts including safety, effectiveness, cost effectiveness, obsolescence and external factors (Table 2). Some focused only on TCPs with little or no health gain and others considered a broad range of factors. No definitive criteria for disinvestment decision-making were identified.

**Table 2:** Examples of theoretical reasons for disinvestment

Reasons for disinvestment	Considerations
Unsafe or harmful (Absolute)	▪ Definitions or operational criteria not provided ▪ Emergency/major safety problems are already addressed through alerts and recalls, no definition or criteria for lower-level safety issues
Less safe (Relative)	▪ Higher rate of the same adverse events ▪ Other adverse events which are thought to be worse (but no guidance/criteria for comparison)
Clinically ineffective (Absolute)	▪ No or very low health gain ▪ No medical indication eg cosmetic procedure
Less clinically effective (Relative)	▪ Lower rate of the same positive outcomes ▪ Other positive outcomes thought to be less desirable (but no guidance/criteria for comparison)
Cost ineffective (Absolute)	▪ Considers effectiveness and cost ▪ Requires threshold, no definition or criteria provided
Less cost-effective (Relative)	▪ Provides less health gain for their cost than alternative ▪ No definition or criteria provided
Outdated, superseded, obsolete	▪ Inferior to more recently introduced TCPs ▪ No definition or criteria provided
External factors	Political decisions, local priorities, rationalisation, organisational capacity and capability

The literature presented the concept of disinvestment from two main perspectives. The first focused on the opportunities for disinvestment in national policy and decision-making processes and was found in government publications. The second was research in academic journals exploring health economics principles or decision-making theory used in disinvestment activities. Most of the research papers were reports of projects to identify a TCP to disinvest or to implement a disinvestment project.

Monash Health staff were not familiar with the term ‘disinvestment’ prior to its introduction in the workshops and interviews. Although the concept was readily understood, participants had no experience of specific definitions or perspectives.

One clear message from the literature, which was also reflected in local responses from Monash Health staff, was that the term ‘disinvestment’ had strong negative connotations and would be a barrier to effective decision-making processes and successful implementation of disinvestment-related change. It was associated with ‘taking away’, ‘cost cutting’, ‘top down interference’ and implied a criticism of current practice. Advice from authors and colleagues was to avoid use of this word. Hence the ‘Disinvestment Project’ became the ‘Sustainability in Health care by Allocating Resources Effectively’ (SHARE) Program.

More recently, questions about the concepts, context, settings, systems, processes and principles for disinvestment have been addressed in systematic reviews [7, 9, 13, 20–24] and other studies [8, 10, 11, 25–29] and more examples of individual projects have been published [20]. No papers discussing the concept of an organisation-wide, systematic, integrated approach to disinvestment in a health service organisation have been identified [30]. The individual elements of this concept have emerged in the current literature with authors recognising the need for systematic [24, 31–39] and integrated approaches [5, 9, 24, 28, 31, 34, 40–45] that are implemented ‘system-wide’ [9, 11, 25].

### What models or methods of disinvestment have been implemented in hospitals or health services?

No theoretical guidance or practical advice for systematically undertaking disinvestment within a health service was identified. The literature review did not find any existing models or proposed methods for an organisation-wide, integrated, evidence-based approach to decision-making [19]. The survey respondents’ research in disinvestment was focused on health economics or policy development and the librarians reported no involvement with disinvestment at all. None had any knowledge or experience to inform development of a systematic organisation-wide approach to disinvestment at the level of a local health service, however all viewed this idea positively. Although the local informants had no experience of disinvestment they were also primarily positive. They identified opportunities and enabling factors within their areas, and highlighted information and resource needs and other potential barriers to disinvestment.

Although there was debate in the literature about processes for disinvestment, there was no consensus or recommendations; and multiple gaps in theoretical knowledge and practical experience were acknowledged. Responses from external experts and local stakeholders were consistent with these findings and also provided additional information. Many issues were framed as questions highlighting the lack of experience in disinvestment. For example, ‘Who has the authority, and the will, to make and act upon decisions about disinvestment?’, ‘Who are the appropriate decision-makers?’ The other points identified in the literature or raised by respondents have been reframed as questions for consistency and all issues are presented in Table 3. The issues raised provide a guide to the information required to develop future models or methods for disinvestment in the local healthcare context.

**Table 3:** Issues to consider in development of an organisational program for disinvestment

Topic	Issues
Organisational and management	▪ How can a systematic evidence-based approach to disinvestment be implemented in a healthcare organisation? ▪ How can disinvestment decisions be integrated into established Strategic and Business Plans ▪ Which is the better approach – ‘top down’, ‘bottom up’ or both? ▪ How to engage and get ‘buy-in’ from clinicians, consumers and other stakeholders ▪ What are the relevant organisational change mechanisms? ▪ What does leadership for disinvestment involve?
Decision-makers	▪ Who has the authority, and the will, to make and act upon decisions about disinvestment? ▪ Who are the appropriate decision-makers? – Existing decision-making bodies or specially convened groups – Composition: policy-makers, managers, clinicians, consumers, technical experts, others – In-house or external ▪ How does the relevant information get to them? ▪ What other agendas do they bring to the decision-making table? ▪ Who has the time, relevant skills and adequate resources to identify, implement and evaluate the required practice changes?
Decision-making	▪ Are all viewpoints equal? ▪ What criteria should be applied to disinvestment decisions and prioritisation? ▪ What is the nature and source of information required? ▪ How do decision-makers become aware of the need to disinvest certain practices? ▪ How are policies and guidance documents used by local decision-makers to allocate resources?
Assumptions	▪ Are generally held assumptions true? For example – ‘Clinicians are reluctant to disinvest’ – ‘Disinvestment is not optimal unless an active intervention is in place’
Skills and resources	▪ What expertise and training is required to make, communicate, implement and evaluate decisions? ▪ What resources are required to source expertise, source information, ‘backfill’ health service staff when participating, and support decision-making, implementation and evaluation processes?
Professional and cultural	▪ What impact will professional boundaries and ‘turf’ issues have on disinvestment activities? ▪ What are the rights and responsibilities of stakeholders? ▪ Different stakeholder views of what is meant by ‘little or no health benefit’ ▪ What is the effect of culture on disinvestment? (authoritative versus consultative, transparent versus hidden) ▪ What are the motives and incentives for disinvestment?
Financial and commercial	▪ What funding is required for disinvestment initiatives and where can it be found? ▪ How can the difficulties inherent in the complex funding arrangements within health services be overcome? ▪ How can savings be measured? ▪ How can savings be reinvested?
Values and ethics	▪ How can transparency of process be ensured? ▪ What is a ‘fair and reasonable’ process? ▪ What are the access, equity and legal considerations? ▪ What is the best way to deal with conflict of interest with commercial entities?
Research and evaluation	▪ What effect will the limited evidence base for some practices have on the process? ▪ How can the lack of tested methods for implementation and evaluation be addressed?

Although the literature has broadened considerably since the initial review was undertaken, a recent review of the current literature was also unable to identify any systematic approaches at the local level [30]. Many of the questions raised remain unanswered [19, 20, 30].

### Where are the opportunities for systematic decisions about disinvestment in a health service?

In establishing the SHARE Program, members of the TCPC took the view that a systematic approach would be better than relying on ad hoc decisions or projects in isolation, and sought to integrate decisions about disinvestment into organisational structures and processes. Since no existing models or methods were identified, a conceptual framework was developed based on the findings from the literature review and the knowledge and experience of Monash Health participants.

A framework is made up of a set of concepts and the relationships between the concepts to facilitate the development of propositions; it provides a frame of reference to organise and focus thinking and assist interpretation [46, 47]. The framework would be used to underpin investigation of the feasibility and utility of the proposed settings and methods for systematic decision-making for disinvestment.

**Fig. 2:**
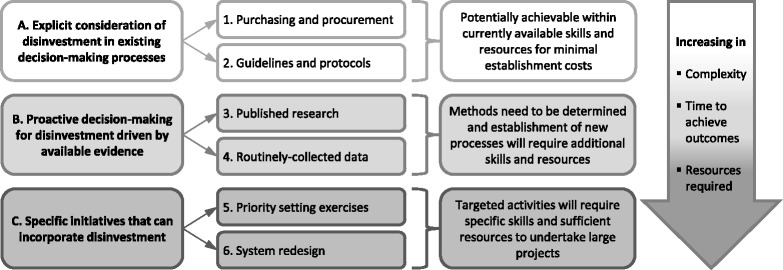
Conceptual framework of potential settings and methods to integrate disinvestment into health service systems and processes

Three mechanisms that provide potential opportunities to introduce disinvestment decisions into health service systems and processes were identified (Fig. 2). They are presented in order of complexity, time to achieve outcomes, and resources required. The first two mechanisms, consideration of disinvestment in existing decision-making processes and proactive use of research evidence and data to drive decisions, were identified by Monash Health participants. The elements of the third mechanism, specific initiatives to consider disinvestment, were identified from the literature.

#### A. Explicit consideration of potential disinvestment in existing decision-making processes

Most health facilities have methods for how they make routine decisions. Two potential opportunities for initiating disinvestment decisions sit within the mechanisms for 1) allocating funding through clinical purchasing and capital procurement and 2) allocating non-monetary resources through guidelines and protocols.

Incorporating considerations for disinvestment into existing systems and processes might be achieved quickly and, once established, delivered with no additional costs.

##### Purchasing and procurement

Monash Health had systems and processes for recurrent purchasing of drugs and clinical consumables and capital expenditure on building and equipment. These were determined by policies and procedures that specified who can make decisions, which criteria are used, how decisions are authorised and other relevant parameters.

This existing structure provides opportunities to integrate prompts, triggers and potentially even mandatory requirements for those making decisions about expenditure to consider disinvestment. For example, prompts and triggers could be implemented in a range of formats such as algorithms, protocols or checklists. Mandatory requirements to consider disinvestment could be implemented as specific directions within purchase orders, explicit decision-making criteria for committees or steps in application processes that require authorisation.

The current literature on resource allocation considers purchasing from a range of perspectives [48–50], but these do not include identifying local disinvestment opportunities.

##### Guidelines and protocols

Guidelines and protocols are designed to inform, direct and standardise clinical and corporate practice. In doing this they also determine allocation of resources for a specific condition, patient group or procedure by stipulating use of drugs or equipment, recommending diagnostic tests, selecting health professional groups, prioritising staff time, specifying referral mechanisms and allocating capacity in clinics, operating rooms and other facilities. There are potential opportunities for disinvestment in all of these activities. Existing decision-making processes for local guidance provide three possible mechanisms to introduce consideration of disinvestment.

Firstly, the process of developing new or revising existing local guidance could be used to identify disinvestment opportunities. Prompts, triggers and mandatory requirements to consider disinvestment in decisions about clinical and organisational practice could be introduced into the processes for document development and authorisation. Document developers and those overseeing the process could be charged with explicitly considering whether any current practices in the content of the guidance could be discontinued.

Secondly, local guidelines and protocols could be used to implement disinvestment decisions. Most guidance documents focus on implementation of effective practices, directing staff to do the things that are known to work. Implementation strategies, such as communication, education and use of tools like clinical paths and checklists, are undertaken to increase staff awareness and compliance with these desirable activities. Local guidance could also be used to recommend removal, reduction or restriction of aspects of current practice which have little or no benefit by incorporating reminders of ‘what not to do’ in the documents and using relevant implementation strategies to emphasise these changes.

Thirdly, potential target areas for disinvestment might also be ascertained through evaluation, audit and review of guidelines and protocols. These activities could routinely identify practices that are not consistent with the best available evidence or are not acceptable to staff or health service consumers. For this to be achieved, evaluators must be made aware of disinvestment concepts and provided with direction and support on how to follow up their findings.

We were unable to find any discussion of local guidelines and protocols being used as a method to identify disinvestment opportunities, however several authors refer to the potential to use guidelines for implementation of disinvestment recommendations [1, 35, 51–53].

#### B. Proactive decision-making about disinvestment driven by available evidence

High-quality evidence to identify potential opportunities for disinvestment is currently available, however it was not routinely accessed by most Monash Health decision-makers. Two sources of evidence that are readily available to health service decision-makers are published research findings and their own routinely-collected data. Monash Health decision-makers often turned to these sources to address problems or to respond to requests to introduce new TCPs, but they did not use it proactively to review current practice, seek opportunities for change or drive priority setting.

The project team noted that before existing evidence can be used proactively to drive decision-making, methods of identifying, capturing, evaluating, disseminating and utilising the information must be investigated. Once effective methods are determined, appropriate infrastructure, adequate resources and high-level skills in EBP and data utilisation will be required for implementation.

##### Published research

There is an increasing body of knowledge about practices that have been demonstrated to be harmful, found to be of little or no clinical benefit, or where a more effective or cost-effective alternative is available. Systems and processes could be developed to take this information directly to decision-makers.

To avoid wasting time and resources considering information that is not trustworthy or does not represent the best available evidence, the committee agreed that only high-quality synthesised information should be used to drive decisions. The project team were aware of publications from sources that require a rigorous process to identify, appraise and summarise all the available evidence systematically and objectively. Systematic reviews, health technology assessments and evidence-based guidelines generally note if there is potential harm or little or no benefit from specific clinical practices.

In addition to these generic sources of evidence, the literature review and consultation with international experts identified rigorous evidence-based publications specifically targeting disinvestment such as the NICE Commissioning Guides [54].

Participants noted that if evidence from the research literature and other publications is used proactively to identify disinvestment opportunities, the addition of information from local data about current use and potential impact of change would be required before decisions to disinvest are made. For example, there may be strong evidence that a particular TCP is not as effective as once thought. This provides a potential target for disinvestment. It should also be a prompt to check whether it is actually current practice within the organisation and that the burden of disease, volume of use, likely outcomes and potential cost of change warrant a disinvestment project or whether the resources would be better used elsewhere.

The range of lists outlining practices that should be discontinued or restricted has subsequently expanded. They are being developed by governments and health agencies [33, 55, 56], commissioners of health services [57], professional bodies [53, 58, 59] and researchers [27, 60]. Given the specific purpose of these ‘low value lists’ and the ease of access to them, it would be tempting to take this information directly to decision-makers. Unfortunately, not all the lists are as trustworthy as the high-quality sources noted above. Some are based on expert opinion only, some from a combination of evidence and expert opinion, and some do not specify methods or provide an explicit definition of ‘low value’. Users of this information may wish to confirm the validity and appropriateness of the claims before acting on the recommendations, in particular the definition being applied and the use of systematic review evidence in the process.

##### Routinely-collected local data

Monash Health routinely collects large amounts of data. Some indicators are required by overseeing authorities, others for in-house purposes, and some are collected for historical reasons that are no longer clear. This is a vastly underutilised source of information.

There is potential to use targeted analysis of routinely-collected data to discover opportunities for disinvestment. Participants proposed three approaches.

The first is to identify areas where a potential disinvestment process might have the greatest impact. Local data could be explored for characteristics such as high volume, high cost, extended length of stay or high rates of adverse events, readmission or re-operation where a change could have a large effect.

The second is to investigate practice variation which could highlight potential disinvestment opportunities. This could be done in-house for comparison between campuses, departments or individuals. If a service does not have an internal equivalent, such as highly specialised programs or high risk patient groups, comparisons can be made with similar services in other organisations. Comparisons of the health service utilisation and patient outcome data described above, as well as differences in rates of prescribing, ordering diagnostic tests or use of specific interventions, could indicate inappropriate or suboptimal practices suitable for disinvestment.

Thirdly, less commonly used data sources such as complaints registers or patient satisfaction surveys could also be explored for trends or emerging themes highlighting inappropriate practices that could be addressed through disinvestment.

In addition to reviewing local data when considering potential disinvestment targets arising from the research literature, participants also noted the reverse; that comparison of current practice against the best available evidence would be required before confirming a decision to disinvest a TCP identified from investigation of local data. For example, if physicians at one campus use twice the amount of a high cost drug than their counterparts at another campus with a clinically equivalent patient cohort, it is likely that one group needs to change their practice. If the physicians at the first campus are overprescribing, this would be an opportunity to reduce overall use, restrict use to a particular indication or replace the drug with a more cost-effective but equally effective alternative. However, it is possible that the group with the higher use actually reflects best practice and the others need to increase their prescribing to achieve optimum patient outcomes. The data only provides an alert to the potential for disinvestment, evidence for best practice from the research literature confirms the need and provides direction.

Two recent studies have used practice variation in national and regional settings specifically to identify ineffective practices and note the potential to do so within local health services, or for health services to benchmark against their counterparts [61, 62].

#### **C. Specific initiatives that can incorporate disinvestment**

Two specific project methodologies with potential to identify targets for disinvestment and implement disinvestment decisions were identified from the literature. Their role in a systematic organisation-wide approach to disinvestment in a local health service has not been explored. Other methodologies for specific project initiatives may also be relevant.

Implementation of priority setting and system redesign initiatives would require very specific skills and sufficient resources to undertake large projects.

##### Economic approaches to priority setting

Much of the literature on disinvestment focuses on use of economic principles to identify and prioritise targets for disinvestment. Specific priority setting exercises can be used to examine resource allocation at the disease, program or health service level. This is done by applying the best available data and making the usually implicit values and opinions that underpin decisions explicit and testable [63]. These methods include examination of current funding levels, how funds are spent and whether reallocation of resources, based on priority setting, would result in a greater benefit. Examples of priority setting models include Program Budgeting and Marginal Analysis, Health Sector Wide Priority Setting, Quality Adjusted Life Year League Tables and Generalised Cost-Effectiveness Analysis [64–66].

Priority setting exercises in the health sector have predominantly been undertaken as research projects by health economists. Translation of these methods from the research setting to routine practice integrated within health service systems and processes could provide additional opportunities to identify and implement disinvestment decisions.

PBMA has subsequently been demonstrated to be effective in making decisions for disinvestment [67, 68] however, although the usefulness of PBMA is acknowledged by decision-makers, they find it difficult to achieve in practice [7, 34, 40]. The major issues are lack of standardised accounting practices, lack of sufficient high quality data to inform decision-making, and lack of time and skills to undertake the process and implement the decisions [7, 11, 31, 34, 40, 69, 70].

##### System redesign

Review of whole systems of care, often referred to as system redesign, may be a potential vehicle for disinvestment. System redesign in health care describes an array of approaches rather than a single technique. A range of methods and tools have been adapted for use in health care including Lean thinking [71], Clinical process redesign [72], Program Logic mapping [73], Plan Do Study Act quality cycle [74] and Failure Mode Effect Analysis [75].

System redesign is a familiar process in health services and offers a well-accepted context to introduce practice change.

More recent publications report that methods used in system redesign have potential to identify disinvestment opportunities and implement and evaluate disinvestment decisions [11, 33, 76]. Using the term ‘system redesign’ is also thought to increase the likelihood of implementation by avoiding the word ‘disinvestment’ [76, 77]. System redesign could be integrated into a systematic organisational approach to disinvestment.

### Limitations

There was no information in the literature or from consultation with international experts in disinvestment about how a local health service might take an organisation-wide, systematic, integrated approach. However there was general agreement about issues that should be considered. Subsequent publications confirm both the validity of this approach and the need to fill these gaps.

Priority-setting exercises and System redesign were already known as methods for change; however the other four conceptual settings arose from brainstorming and extrapolating from issues identified in the literature and local consultation. In the absence of evidence, the framework of three pairs of opportunities to initiate disinvestment decision-making was primarily developed from knowledge of healthcare services and logical thinking. There may be other settings that provide opportunities for disinvestment that were not included in this framework. Some of the settings in this framework may not be applicable in other health services, and settings identified elsewhere may not be applicable to Monash Health.

The study samples were purposive but small, limiting the generalisability to other health services. However the subsequent SHARE activities exploring the feasibility and utility of these early proposals include extensive stakeholder consultation involving all health professional groups, managers, policy-makers and consumers [78–83].

Some countries, states/provinces or regions have more centralised decision-making and resource-poor countries may not have the same systems and processes or the capacity or capability to implement any proposed innovations, also limiting the generalisability.

## Conclusion

There is no common terminology. There are multiple definitions for disinvestment based on a range of different concepts and numerous alternative terms to convey the same concepts. However there is one notably consistent message; the word ‘disinvestment’ has negative connotations and may be a barrier to effective decision-making processes and successful disinvestment outcomes.

No theoretical guidance or practical advice for an organisation-wide approach to disinvestment at the local health service level was identified. Further research in this area is needed.

The six concepts captured in the framework create potential opportunities to initiate disinvestment activities in a systematic, integrated approach that can be applied across a whole organisation using transparent, evidence-based methods. Incorporating considerations for disinvestment into existing decision-making systems and processes might be achieved quickly with minimal cost; however establishment of new systems requires research into appropriate methods and provision of appropriate skills and resources to deliver them.

## Additional file

Additional file 1: Methods (PDF 287 kb).

## Abbreviations

CCE: Centre for Clinical Effectiveness
EBP: Evidence Based Practice
MH: Monash Health
NICE: National Institute for Health and Clinical Effectiveness
SHARE: Sustainability in Health care by Allocating Resources Effectively
TCPC: Technology/Clinical Practice Committee
TCPs: Technologies and clinical practices
