# Supplementary material for: Sustainability in Health care by Allocating Resources Effectively (SHARE) 2: identifying opportunities for disinvestment in a local healthcare setting
Source: BMC Health Serv Res. 2017 May 5;17:328. doi: 10.1186/s12913-017-2211-6 (PMC5420107; doi:10.1186/s12913-017-2211-6)
Supplement: Additional file 1: — Methods (PDF 287 kb). [file 12913_2017_2211_MOESM1_ESM.pdf]

Table 1: Summary of methods

DATA COLLECTION
**Literature review**
**Aim:** To understand the concepts related to disinvestment and their implications in a local health service and to ascertain examples of existing decision-making systems and processes in this setting.<br>**Search terms:** Medical Subject Headings (Health Care Rationing, Resource Allocation, Health Priorities and Health Services Needs and Demand) and Text words (disinvestment, decommissioning, defunding, resource release, allocation, reallocation, hit list, ineffective services, low value services, wish list, exclusions, priority setting, program budget marginal analysis, PBMA, resource scarcity, rationing, invest to save) were used with truncations appropriate to the databases utilised. The search strategy was iterative with new terms added as they were identified.<br>**Sources:** Medical databases (Ovid Medline, All EBM Reviews, EMBASE, Cochrane Library), the internet (via the Google search engine) and guideline websites. These methods were supplemented by follow up of reference lists in key publications and searches for publications by identified authors in the field.<br>**Inclusion criteria:** English language publications including guidelines, reviews, research studies, technical reports or policy documents that addressed the issue of disinvestment from a conceptual (terminology, definitions and operational criteria) or policy perspective.<br>**Data Collection and Analysis:** Inclusion, exclusion and appraisal criteria were established a priori. Publications that did not meet the criteria were excluded on review of title and abstract. When a decision could not be made based on abstract alone, full text was retrieved. Critical appraisal appropriate to study design was planned but no research studies were identified.<br>**Search results:** Nineteen documents met the inclusion criteria. These were mainly publications providing a statement of the policy context, the rationale or need for disinvestment and/or a critique of existing processes. A small number of case reports were included but no research studies were identified.<br>**Synthesis:** Information from articles which met the inclusion criteria was summarised based on content relevant to the themes of conceptual and policy perspectives determined a priori.<br>Full details are in the review publication [19].
**Interviews with members of the Technology/Clinical Practice Committee**
**Aim:** To identify opportunities for disinvestment in existing or potential decision-making settings and consider implications for disinvestment in the Monash Health setting<br>**Participants:** The Executive of the Technology/Clinical Practice Committee (TCPC), the initiators of the SHARE Program, included an executive director, medical director, clinical program director and research director.<br>**Data collection:** Semi-structured group and individual discussions were conducted using prompts based on the two aims; discussions were documented in the minutes.<br>**Analysis:** Responses were collated and added to findings from the other sources which were then analysed thematically by content analysis.<br>**Response rate:** All 4 informants participated.<br>**Representativeness of sample:** Participants represented senior decision-makers from a range of contexts
**Survey of external experts**
**Aim:** To ascertain unpublished experiences or examples of models or methods for disinvestment in the local healthcare setting.<br>**Participants:** 1) Disinvestment researchers initially identified from publications and websites about disinvestment and subsequently using a snowballing technique based on feedback from respondents. 2) Subscribers to the Health Technology Assessment (HTA) email list.<br>**Design:** The organisation-wide systematic approach to disinvestment proposed in the SHARE Program was described in an email. Participants were asked if they had experiences of disinvestment in the local healthcare context that could inform Monash Health decision-making, any unpublished reports or other documents on this topic, and current or planned research in this area.<br>**Data collection:** Responses were received by return email.<br>**Analysis:** Responses were collated and added to findings from the other sources which were then analysed thematically by content analysis.<br>**Response rate:** Eleven of the 14 researchers and four health librarians from the HTA list (denominator unknown) responded to the survey.
**Structured workshops with the SHARE Steering Committee**
**Aim:** The workshops had several aims, the component reported in this paper relates to identification of opportunities for disinvestment in existing or potential decision-making settings.<br>**Inclusion criteria:** Senior decision-makers at Executive and Director level and health service consumers<br>**Sampling:** Convenience sampling was used to include members of the SHARE Steering Committee comprising Executive Directors (Medical, Nursing, Support Services), clinical Program Directors (Medical, Nursing, Allied Health, Pharmacy, Diagnostic Services), Committee chairs (Technology/Clinical Practice, Therapeutics, Human Research and Ethics, Clinical Ethics), Directors of non-clinical services (Information Services, Clinical Information Services, Procurement, Biomedical Engineering, Research Services), Legal counsel and two consumer representatives. Two representatives from the Department of Human Services Technology Division also participated.

Approach: Workshops were conducted at scheduled Steering Committee meetings.

Design: Workshops were based on the first two steps in the SEAchange model for evidence-based change [16]; identifying the need for change and developing a proposal for change. Presentations outlining the background and aims of the workshops were made by the project team, discussion was structured around the questions to be addressed and decisions were based on consensus. Questions included:

Workshop 1: Where and how are decisions made, documented, communicated, implemented and evaluated and what are the related system issues? Where is change required? Why? What is the problem? How can the need for change be measured? What are the factors enabling sustainability of the current system? How is it integrated?

Workshop 2: What existing systems/processes work well that we could maintain as they are, should be ceased, could be kept but require improvement? What new systems/processes should be introduced? What structures, skills, resources, commitment and leadership are required? Are they available? If not, how can they be obtained? What existing systems can be utilised? What is the solution to the problem? What are the options? What is known about best practice in this area? What is required to ensure sustainability of the proposed system? How can it be integrated?

Data collection: Participants completed prepared worksheets and discussed the findings. Discussion and decisions were documented in minutes.

Respondent validation: Minutes were approved at the following meeting.

Analysis: Data from the worksheets and findings from the discussion were collated and organised in MS Word and Excel. Emergent themes were identified by framework analysis.

Response rate: Thirteen members participated, 9 attended the first workshop, 11 attended the second, and some non-attenders also completed the worksheets.

Representativeness of sample: A range of senior decision-makers were represented at each workshop, plus representatives from the state health department.

Interviews with key local informants

Aim: To test preliminary thoughts regarding direction of the SHARE Program with front line staff and consumers

Participants: Six participants selected purposefully and pragmatically to seek the views of a range of Monash Health decision-makers: the five senior clinicians were program directors and department heads representing medicine, surgery, nursing, allied health and diagnostic services and the consumer representative had experience on committees that made organisation-wide decisions.

Interview schedule

Disinvestment: Have you heard about the concept of disinvestment?

Potential settings/methods: Are you aware of any of these? Do you do any of these sorts of things? What could you do in your Unit? What could be done in your Program/Division? What could be done by your colleagues eg referrers? Any opportunities for quick wins? Incentives to change? Barriers to change? Potential to link into advanced trainee projects?

Research evidence: What information do you use? Where from? How do you access it? What do you do with it? Could you use more? What would you like? How would you like it? What would you do with it?

Local data: Do you use Monash Health data? How? What for? Do you use external data? What? How? What for? Could you use it? How?

General discussion: How could we get wider feedback? Should we survey, etc? Should this be driven top down or bottom up? Would you be interested in piloting something?

Data collection: Structured interviews were conducted using the interview schedule above; one CCE staff member attended and took notes.

Analysis: Responses were collated and added to findings from the other sources which were then analysed thematically by content analysis.

Response rate: All 6 informants participated.

Representativeness of sample: Interviewees represented senior decision-makers from a range of contexts

DELIBERATIVE PROCESS

Structured workshop with SHARE Steering Committee

Aims: To review and refine draft proposals, frameworks and plans and make final decisions.

Participants: SHARE Steering Committee members including Executive Directors (Medical, Nursing, Support Services), Program Directors (Medical, Nursing, Allied Health, Pharmacy, Diagnostic Services), Committee chairs (Technology/Clinical Practice, Therapeutics, Human Research and Ethics, Clinical Ethics), Managers (Information Services, Clinical Information Services, Procurement, Biomedical Engineering, Research Services), Legal counsel and two Consumer representatives.

Design

- Provision of pre-reading materials and/or workshop presentation of background, issues to consider, draft proposals, etc
- Agenda including points for discussion and decisions required
- Decisions made by consensus
- Documentation of discussion, decisions and actions in minutes
